# The Impact of School Climate and School Identification on Academic Achievement: Multilevel Modeling with Student and Teacher Data

**DOI:** 10.3389/fpsyg.2017.02069

**Published:** 2017-12-05

**Authors:** Sophie Maxwell, Katherine J. Reynolds, Eunro Lee, Emina Subasic, David Bromhead

**Affiliations:** ^1^School of Education, RMIT University, Brunswick, VIC, Australia; ^2^Research School of Psychology, Australian National University, Canberra, ACT, Australia; ^3^Psychology, School of Psychological and Clinical Sciences, Charles Darwin University, Darwin, NT, Australia; ^4^School of Psychology, University of Newcastle, University Drive, Callaghan, NSW, Australia; ^5^Student Engagement and Wellbeing, Australian Capital Territory Education Directorate, Braddon, ACT, Australia

**Keywords:** academic achievement, school climate, school identification, social identity, student and staff/teacher perceptions, multilevel analysis

## Abstract

School climate is a leading factor in explaining student learning and achievement. Less work has explored the impact of both staff and student perceptions of school climate raising interesting questions about whether staff school climate experiences can add “value” to students' achievement. In the current research, multiple sources were integrated into a multilevel model, including staff self-reports, student self-reports, objective school records of academic achievement, and socio-economic demographics. Achievement was assessed using a national literacy and numeracy tests (*N* = 760 staff and 2,257 students from 17 secondary schools). In addition, guided by the “social identity approach,” school identification is investigated as a possible psychological mechanism to explain the relationship between school climate and achievement. In line with predictions, results show that students' perceptions of school climate significantly explain writing and numeracy achievement and this effect is mediated by students' psychological identification with the school. Furthermore, staff perceptions of school climate explain students' achievement on numeracy, writing and reading tests (while accounting for students' responses). However, staff's school identification did not play a significant role. Implications of these findings for organizational, social, and educational research are discussed.

## Introduction

Effective teaching and learning is the result of complex group and psychological processes. However, the precise organizational factors and psychological mechanisms behind these processes are still under investigation. Identifying the means to improve students' learning outcomes remains the subject of continuous academic inquiry and a key objective of government and international bodies. As a result of this interest, an immense body of work centerd on the construct of “school climate” has emerged. School climate refers to social characteristics of a school in terms of relationships among students and staff/teachers, learning and teaching emphasis, values and norms, and shared approaches and practices (Anderson, 1982; Moos, 1987; Thapa et al., 2013). Among other factors, empirical evidence has confirmed that school climate is powerful in affecting students' academic achievement (Brand et al., 2008; Chen and Weikart, 2008; Collins and Parson, 2010). However, the extent to which *both of student and staff perceptions of school climate* influence student achievement is less clear. Furthermore, the precise psychological processes underpinning the climate-achievement link requires further investigation.

Seeking to fill these gaps, the current research examines the impact of student and staff perceptions of school climate on students' achievement. Very few studies have investigated both groups' perceptions of school climate in relation to academic achievement and even fewer using a robust, national, standardized measure to assess achievement. The present research also offers a theoretical analysis of the psychological processes underlying this relationship, using the social identity approach (Tajfel and Turner, 1979; Turner et al., 1987). This analysis builds on work that has applied the social identity approach to various staff and student outcomes (Bizumic et al., 2009; Turner et al., 2014; Reynolds et al., 2017) and has relevance for school-based interventions directed at improving school outcomes.

In the following sections, the construct of school climate is described, along with the links between (a) student perceptions of school climate and students' academic achievement and (b) staff perceptions of school climate and students' academic achievement. Next the theoretical framework, the social identity approach is introduced. Finally, some methodological challenges confronting researchers in this field are described.

## What is school climate?

The school climate construct is complex and multi-dimensional. It has been described as the unwritten personality and atmosphere of a school, including its norms, values, and expectations (Brookover et al., 1978; Haynes et al., 1997; Petrie, 2014). Further, it has been described as the “quality and character of school life” (Cohen et al., 2009, p. 182). Importantly, rather than concerning administrative or physical attributes of the school (e.g., teachers' salary or schools' physical resources), school climate research hones in on the psychosocial school atmosphere, and the inter-group interactions that affect student learning and school functioning (Johnson and Stevens, 2006; Lubienski et al., 2008; Reyes et al., 2012).

School climate is a leading predictor of students' emotional and behavioral outcomes. It affects students' adaptive psychosocial adjustment (Brand et al., 2008), mental health outcomes (Roeser et al., 2000; Brand et al., 2003) and self-esteem (Way et al., 2007). School climate also influences students' behavior, such as rates of bullying and aggression (Espelage et al., 2014; Turner et al., 2014), student delinquency (Gottfredson et al., 2005), and alcohol and drug use (Brand et al., 2003). Finally, and of particular relevance to this research, school climate perception has also been found to affect students' academic achievement (Brookover et al., 1978; Brand et al., 2008).

## The challenge of defining and measuring school climate

The multiplicity of definitions for school climate has led to confusion and hindered research progress (Hoy and Hannum, 1997; Thapa et al., 2013; Ramelow et al., 2015; Wang and Degol, 2015; Lee et al., 2017). This lack of definitional consensus has meant that school climate is measured inconsistently (Thapa et al., 2013). Various scales have been used, with their different sub-scales flowing from different articulations of the construct. Despite this limitation, three sub-factors of the construct (Moos and Moos, 1978) are clearly represented in the literature and school climate scales. (1) School's academic emphasis as personal growth or goal orientation; “the extent to which a school is driven by a quest for academic excellence” (Hoy et al., 1991, p. 71); (2) interpersonal relationships within a school, which are judged by their quality and consistency (Haynes et al., 1997); and (3) shared norms, goals, and values; the common understanding of accepted and endorsed behavior (Frederickson, 1968). These defining sub-factors have brought some conceptual clarity to the construct.

The assessment of school climate involves asking particular groups of interest to report their perceptions. These groups' perceptions include parents' (Esposito, 1999), students' (Fan et al., 2011), principals' (Brookover et al., 1978), and teachers' (Johnson and Stevens, 2006; Brand et al., 2008; Bear et al., 2014). Perspective matters because each group may perceive school climate differently. Often, though, only one group's perceptions have been assessed, usually students in most studies.

## Students' perceptions of climate and academic achievement

Variance in achievement beyond individual factors and socio-economic status has consistently been explained by students' school climate ratings (Hoy and Hannum, 1997; Brand et al., 2008; Collins and Parson, 2010). Brookover et al. (1978) conducted a seminal study establishing this student-climate-achievement link. The authors tested the effect of students' perceptions of school climate on mean school achievement in three samples of racially diverse elementary schools. They found that school climate explained a significant amount of the between-school variance in mean school achievement and that the strength of the relationship was similar to that explained by economic status (SES) and ethnicity.

Subsequent research supports these findings (Goddard et al., 2000b; Heck, 2000; Thapa et al., 2013). For example, Hoy and Hannum (1997) and Tschannen-Moran et al. (2006) found that positive school climate was associated with students' academic achievement, after controlling for SES. Contrastingly, a negative school climate has been found to reduce student participation in school activities and student learning (Chen and Weikart, 2008). This climate-achievement relationship appears to be robust for students across different grades, backgrounds, and cultures (Gregory et al., 2007; Jia et al., 2009). It also appears to endure for years (Hoy et al., 1998), which has been further supported by longitudinal studies (e.g., Brand et al., 2008).

Various sub-factors of school climate have been found to exert a powerful impact on academic achievement. For example, academic emphasis (Hoy and Sabo, 1998; Goddard et al., 2000b), academic optimism (Smith and Hoy, 2007), and strong teacher-student relationships (Crosnoe et al., 2004; Tschannen-Moran et al., 2006) have been found to be particularly influential. In particular, student-teacher relationships effectively work as a protective factor for school adjustment including academic achievement as well as conduct and behavioral problems, especially for adolescents transiting from middle school to high school (e.g., Longobardi et al., 2016). However, many of the reviewed studies are limited because of how they measured academic achievement. Many have relied on regional or state-wide tests and unstandardized measures (e.g., self-reported performance or grade point average, [GPA]). Although various studies have used standardized literacy and numeracy assessment data (e.g., Goddard et al., 2000b; Sweetland and Hoy, 2000; Tschannen-Moran et al., 2006; Brand et al., 2008), studies using standardized *nation*-wide tests are limited.

## Staff perceptions of school climate and academic achievement

While studying the climate-achievement link from the student perspective is illustrative, the staff perspective is also relevant (Fisher and Fraser, 1983; Johnson et al., 2007; Liu et al., 2014). Measuring staff perspectives of school climate is important for several reasons. First, discrepancies have been found between students' and teachers' perceptions. Teachers' ratings are more sensitive to classroom level factors and students are more sensitive to school-level factors (Mitchell et al., 2010; Wang and Eccles, 2014). Teachers also rate teacher-student relations more positively than students do (Raviv et al., 1990). Second, and importantly, teachers have the largest impact on student learning out of all school reform initiatives (Heck, 2000; Lindjord, 2003; Schacter and Thum, 2004). Therefore, measuring staff perceptions might expose areas for reform and intervention.

A relatively small pool of literature measures the effect of staff's perceptions of school climate on student outcomes. These links have often been vague, with methodological challenges undermining the research to date. The dominant focus has been how staff perceptions of school climate affect *staff's* functioning (Heck, 2000). For example, staff perceptions have been measured against staff well-being (Boyd et al., 2005; Grayson and Alvarez, 2008), staff morale and job satisfaction (Ma and MacMillan, 1999; Collie et al., 2012). The impact of staff perceptions on *student* outcomes, such as student achievement has been explored to a much lesser extent. Nevertheless, there is a general trend observed in the relationship between staff climate perception and student achievement.

Early studies highlight that staff perceptions of the schools as a work environment and expectations of students affect student outcomes (Moos, 1987; Esposito, 1999). More recent studies support these findings. For example, Johnson and Stevens (2006) found teachers' perceptions of school climate had a positive relationship with fourth graders' scores on standardized tests using structural equation modeling. However, a drawback of their design was their use of aggregated mean scores for staff perceptions and student academic performance by school. This design assumes there is no difference within schools. By ignoring and compressing individual variation, important statistical information is also lost (Hox, 2010) and standard error estimates may be incorrect (Garson, 2013). This methodological approach is a common limitation in educational research, and will be further described later in this introduction.

A more comprehensive study exploring the impact of staff climate perceptions on student achievement was carried out by Brand et al. (2008). There were three particularly relevant findings. First, teachers' school climate perceptions were significantly associated with eighth graders' reading and mathematics scores. Second, teachers' reports of students' achievement orientation were significantly correlated with students' mathematics achievement and reading performance. Third, teachers' climate perceptions were significant predictors of less robust measures of achievement, such as GPA and students' academic efficacy. Their statistical design was strong, as they used hierarchical linear modeling to control for the nested structure of the data. The authors also controlled for student's SES, used a longitudinal design (3-year period) and large samples with up to 114, 240 students from 243 schools.

Additionally, the authors used a paired school climate scale to measure student and teacher perceptions. They then compared the effect of teacher perceptions to the effect of student perceptions on the same variables. Out of all aspects of teachers' and students' perspectives of school climate, achievement orientation emerged as the strongest predictor of student achievement. Furthermore, schools had higher achievement levels when teachers perceived positive student-student relationships (“peer sensitivity”) and lower levels of disruption, which was not the case with student perspectives.

Although there is less literature exploring the relationship between staff perceptions of school climate and achievement (compared with literature from the student perspective), there is general support showing that staff perceptions of school climate predict student achievement. However, the way in which school climate perception comes to affect student achievement is still to be explored.

## How does school climate perception affect student achievement?

Explaining precisely how school climate perception comes to affect student outcomes has been a challenge for researchers. In any case, various theories have been put forward (see Wang and Degol, 2015 for a comprehensive review), including social cognitive theory, self-determination theory, and bio-ecological theory. However, the social identity approach offers an alternative and integrative analysis, which will be adopted in the current research.

Social cognitive theory has been a particularly popular theoretical explanation for the climate-achievement link as it relates to students and staff (Bandura, 1993, 1997). Authors have suggested that students need collective efficacy to activate the influence of the school climate, in particular for the aspect of academic press, on their achievement (Hoy et al., 2002). This approach has also been applied in explaining the impact of staff perspectives on student achievement (Hoy and Woolfolk, 1993; Goddard et al., 2000a). For example, Caprara et al. (2006) found that teachers' self-efficacy beliefs were significantly related to students' academic achievement. Goddard et al. (2000a) additionally found that collective teacher efficacy significantly predicted students' reading and mathematics performance. Specifically, the authors found that a “one unit increase in a school's collective teacher efficacy score” was related to increase of “more than 40% of a standard deviation in student achievement” (p. 501).

Self-determination theory has also been widely applied (Deci and Ryan, 1985). Authors have proposed that students and staff need to meet the psychological basic needs of relatedness, competence, and autonomy in order for students to achieve (Connell and Wellborn, 1991; Roeser et al., 1998; Reeve, 2012; Taylor et al., 2014). Bronfenbrenner's bio-ecological theory has also been investigated through analyzing how the layers of the environment (e.g., individual, family, and school) affect student learning (Bronfenbrenner, 1979, 1986; Rosenfeld et al., 2000; Stewart, 2007; Hampden-Thompson and Galindo, 2017).

The theories have much to offer in understanding the climate-achievement link, in terms of intrapsychic individual psychology. Yet, exploring a whole school approach and group dynamics in a school may offer further theoretical and practical implications. Indeed, specific theories within social psychology that focus on group-level processes provide a novel perspective to explain the effect of school climate on achievement. Hence, the social identity approach is put forward as an integrative theoretical explanation for this school climate-achievement link.

### Background to the social identity approach

The “social identity approach” consists of social identity theory (Tajfel and Turner, 1979) and self-categorization theory (Turner et al., 1987). The key point of the social identity approach is that a group, system or organization (e.g., school) influences individual behavior (e.g., student or staff member) when an individual feels psychologically part of that group, system, or organization (Tajfel and Turner, 1979). Membership to these higher-level systems is not defined by external criteria (e.g., the category of student, label of staff member, or any other demographic characteristic). Rather, it is defined by a feeling of psychological membership, identification, and connectedness.

The social identity approach makes an important distinction between a personal identity and a social identity (“I” or “me” vs. “we” or “us”; Turner et al., 1987). When an individual finds a group *psychologically* meaningful (becoming “we” or “us”), the group's values and needs become normative and are integrated into personal ones (Turner et al., 1994). The process of social identification entails members becoming motivated to achieve the group's goals and putting more effort into ensuring these goals are realized (Haslam et al., 2000). In other words, the individual's psychological connection with the group triggers the influence of organizational factors on their behavior and makes them more likely to act in alignment with the group's norms and values (Turner, 1985; Turner and Reynolds, 2011).

In the school context, norms, values, and beliefs of the “school” group are embodied in the school climate construct. A central goal of the school as a group is often to have a strong academic emphasis, supportive staff-student relations, and shared values and approach (factors which are conducive to successful student learning) (Bizumic et al., 2009; Reynolds et al., 2017). It is possible to conceptualize school climate as the *facilitator* of students' and staff's identification and school identification as the psychological *process* through which school climate comes to affect their behavior.

Students' school identification might affect their academic performance in the following way. If the school climate is positive and supportive, and this, in turn, facilitates the student to identify with the school as a salient group, then the student is more likely to reflect and embed the school values and norms, focusing on learning and achievement, with their behavior (Reynolds et al., 2017).

Along these lines, Reynolds et al. (2017) found that the relationship between students' school climate perceptions and students' numeracy and writing scores was fully mediated by students' school identification. However, the measure of school climate was limited in their study and featured only one general dimension of school climate, which was shared values and approach.

More broadly, related concepts to social identification have also been captured by the educational literature, and studied in relation to student outcomes. For example, connectedness, student-school bonding, attachment, and sense of belonging to school have been studied (Osterman, 2000; Libbey, 2004; Blum, 2005; Vieno et al., 2005; Waters et al., 2009). One particularly relevant study for students found the relationship between school climate and students' conduct problems was mediated by students' school connectedness (Loukas et al., 2006). School belonging also was a critical variable, especially for multiracial modeling of student achievement (Burke and Kao, 2013; Hernández et al., 2014; Gummadam et al., 2016).

The social identity approach can also be applied to explain the link between staff school climate perception and student achievement (Reynolds et al., 2017). The outcome of interest in the present research is *student* achievement. Thus, the relevant outcome is the behavior of the *students*. Therefore, it seems illogical to also propose *staff* school identification as a psychological mediator of the students. However, there remains different reasons to assume that staff school identification could play an important role. Rather than mediate, staff school identification might moderate the influence of their climate perception on student achievement. That is, the level of staff's psychological membership to the school might adjust the impact of school climate on students' achievement. For example, when staff strongly identify themselves with the school, staff might be more motivated to strive for better academic results from their students in the classroom and dedicate more effort to fostering supportive relations with students. These behaviors are conducive to students' academic engagement, which may translate to students' improved student achievement, *only when* staff social identity as a school member is high. That is, the strength of the path from staff school climate perception to student achievement would be dependent on the level of staff school identification, as a regulator. If staff social identification is weak, then the impact of their school climate perception on student achievement may be far weaker posing different impact strength from for the case with higher staff school identification.

Unlike the application of the social identity approach to students, this specific theoretical proposition with respect to staff school identification has not been directly investigated. However, a link between staff behavior (more broadly) and student performance has been well-established. For example, Mohammadpour (2012) found after controlling for some student and school factors, teacher emphasis on homework had a significant association with student achievement. MacNeil et al. (2009, p. 155) also emphasized the importance of teacher morale and motivation for student outcomes, finding that “highly motivated teachers have greater success in terms of student performance.” Teachers and administrators' feeling of a sense of school cohesion influenced students' academic achievement (Stewart, 2007). Additionally, teacher empowerment was found to be a significant predictor of students' results on standardized tests (Sweetland and Hoy, 2000). The important point to distill from these studies is that psychological phenomena applying to staff have been found to affect the behavioral outcomes (specifically, achievement levels) of students.

Importantly, most of these studies have only looked at certain variables as *predictors* of students' academic achievement, and not as psychological mechanisms or moderators. This study takes a novel approach by proposing that students' school identification is a mediator and staff's school identification is a moderator of the relationship between their perceptions of school climate and student achievement. This approach is important because “social identity processes not only help explain student behavior at school but point to pathways that can be used to shape it” (Reynolds and Branscombe, 2015, p. 171). A better understanding of the underlying processes may be especially informative in designing effective and efficient interventions to improve achievement outcomes.

## The current study

The extant literature has demonstrated that students' and members of staff's ratings of school climate have a significant impact on students' academic outcomes. Nevertheless, there a number of gaps and issues in this body of work to be addressed. First, although some parallel measures have assessed both students' and staff's school climate perception (e.g., Brand et al., 2008), little is known about whether staff school climate perception plays a significant role when student perceptions and other covariates are taken into account in a single statistical model. Second, many studies of academic achievement have used unstandardized tests and single-informant school climate perspectives. Third, the nested hierarchical inter-correlations of student and staff data within schools has often been ignored, which can be addressed through the use of multilevel modeling (D'haenens et al., 2010; Wang and Degol, 2015). Finally, there is room for theoretical and empirical exploration of the psychological processes accounting for the climate-achievement relationship.

In aiming to address these gaps, the present study proposes MLM procedures, standardized achievement data and multi-informant data (student and staff perceptions and educational records) to examine both the impact of student and staff perceptions of school climate on students' standardized literacy and numeracy tests. The models should also control for demographic variables including gender, parental education, school size, and SES. Further, it will expand our knowledge and inform school reformers to investigate whether those relationships operate as a function of students' and staff's psychological identification with the school climate, i.e., “school identification.”

In the present study there are three informant sources are integrated in a single study design; survey responses both from staff and students, as well as NAPLAN data and demographic information from education records. The study employs MLM methods to address some of the problems suffered by past studies of aggregation bias, heterogeneity of regression, and increased errors in parameter estimation (Bryk and Raudenbush, 1988; Tabachnick and Fidell, 2014).

Core covariates are also included in the analysis. Students' gender is included due to its known effect on academic achievement (Marsh et al., 2005; Hinnant et al., 2009). Male students have an advantage on numeracy tasks whereas females may have an advantage in verbal information tasks (Halpern and LaMay, 2000; Ma and Klinger, 2000). This gender difference has been reflected in NAPLAN data for 2008–2013, where males have performed consistently better in numeracy tests (Australian Curriculum Assessment Reporting Authority, 2008-2015). The level of education of students' parents is also included, as it is also known to affect student achievement (Davis-Kean, 2005; Senler and Sungur, 2009).

School covariates are included, namely, the SES of the whole school (Caldas and Bankston, 1997, 1999; Johnson et al., 2001; Perry and McConney, 2010) and school size (Lee and Loeb, 2000; Ma and Klinger, 2000). These individual factors (students' gender and the educational level of their parents) and school factors (SES of the school and school size) are controlled in order to measure the impacts of school climate perception and identification on NAPLAN results more clearly.

While a similar study measured the impact of students' school climate perception and school identification on NAPLAN results (Reynolds et al., 2017), the present study uses a significantly larger sample size (2,257 students in 17 secondary schools) compared to their study (340 students in 2 schools). Compared with their study, a more fully developed version of School Climate and School Identification Measurement Scale (Lee et al., 2017) is used. Furthermore, multilevel modeling is employed and staff perceptions are additionally investigated.

The current study also explores the role of school identification in the climate-achievement relationship. Students' school identification is modeled as a mediator of the link between students' perceptions and their achievement. Mediation models (MacKinnon, 2008; Hayes, 2009) were tested using the following paths; (1) from school climate to school identification, (2) from school identification to achievement scores, and (3) the indirect path from school climate to achievement scores via school identification. In contrast, staff's school identification is modeled as a moderator. It is hypothesized to interact with the relationship between staff school climate perception and student achievement, such that the level of staff school identification changes the strength and nature of the potential relationship between staff perceptions and student achievement.

**Figure 1:**
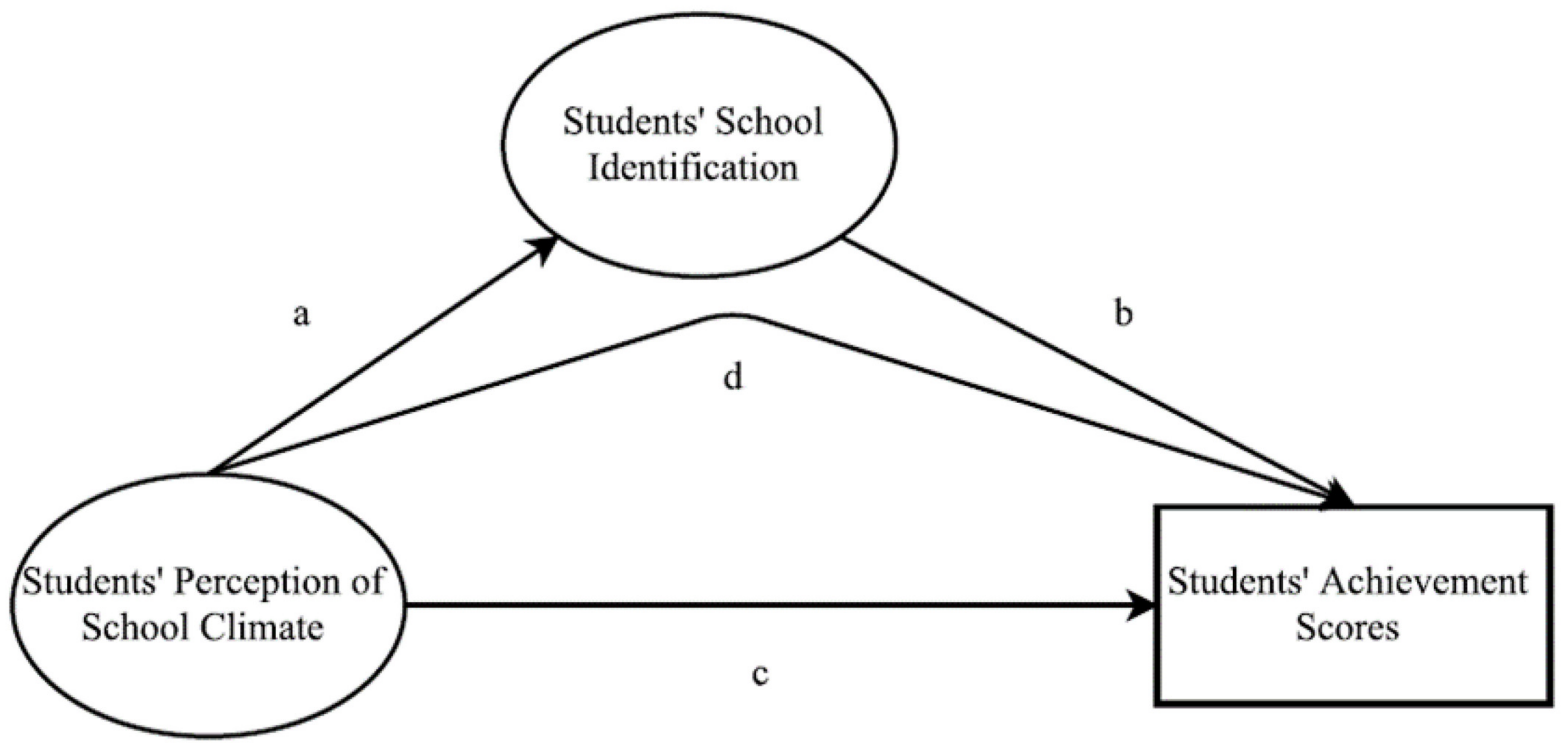
Conceptual model for Hypothesis 1: c = the direct path from students' perceptions of school climate to students' NAPLAN results will be positive and significant. Conceptual model for Hypothesis 2: a = students' positive school climate will predict stronger school identification among students, b = the path from students' school identification to students' NAPLAN results will be significant and d = the indirect path from school climate to student achievement scores via school identification will be positive and significant.

Accordingly, the current research proposes that after controlling for demographic factors, students' school identification will mediate the impact of student's school climate perception on academic achievement. More specifically, corresponding to Figure 1, positive school climate perception will predict stronger school identification among students (*a*) that in turn predict higher achievement scores (*b*). The indirect path from school climate to student achievement scores via school identification will be positive and significant (*d*).

**Figure 2:**
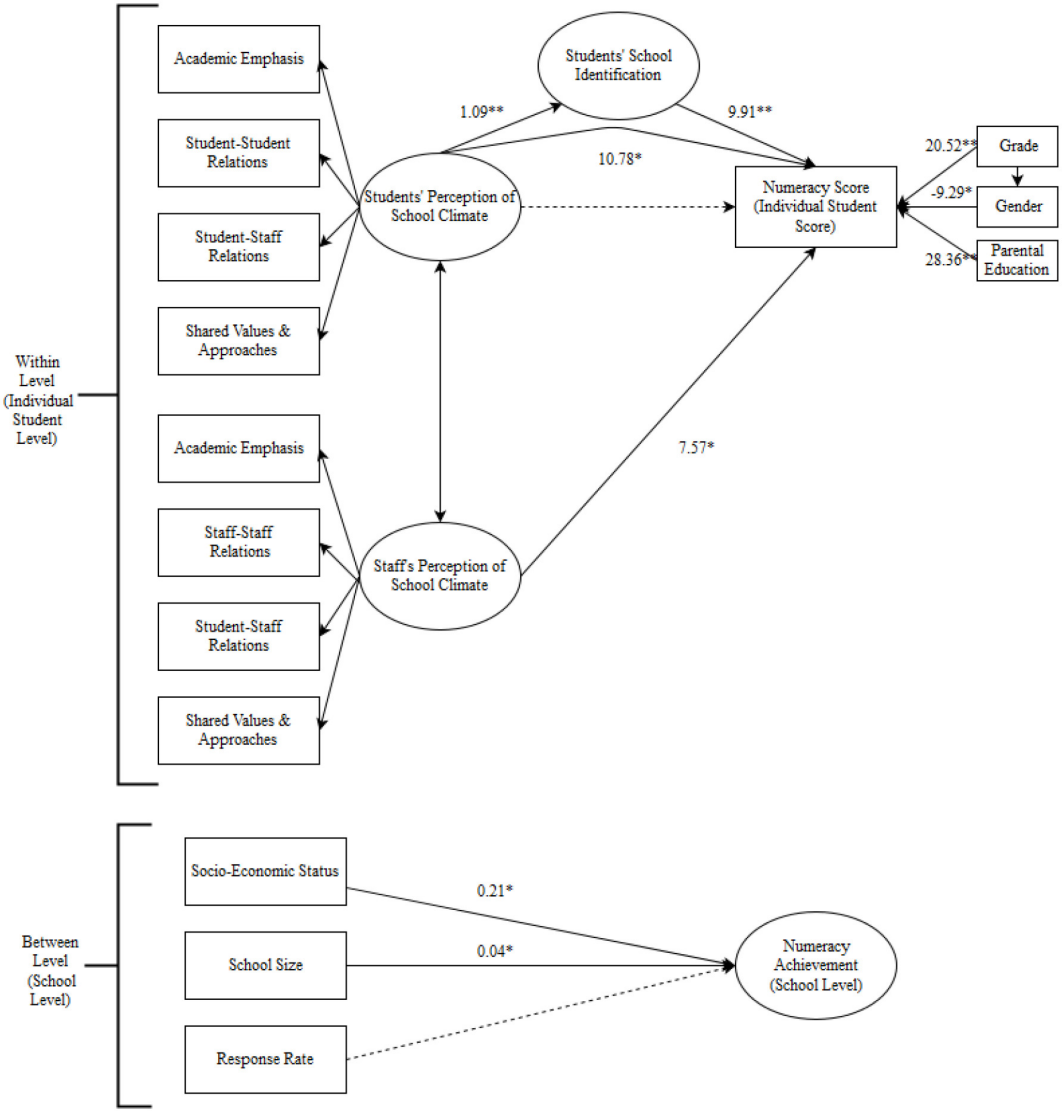
Model 5: Multilevel SEM of numeracy scores with student and staff school climate perception predictors, a mediator of student school identification, and demographic covariates at the student and school level. Error terms, correlations, and related coefficients are ommitted for simplicity. Gender: Male = 0, Female = 1; Parental education: below university degree = 0, university degree or higher = 1. Coefficients are unstandardized. ^*^p < 0.05; ^**^p < 0.01.

We also hypothesize that Staff perceptions of school climate will predict students' higher levels of academic achievement. Furthermore, staff's school identification will moderate the impact of their school climate perception on students' academic achievement. A high level of school identification amongst staff will explain a stronger impact of staff perception of school climate on students' academic achievement, whereas a low level of school identification will explain a weaker impact of staff school climate perception on students' academic achievement (Refer to Figure 2).

## Methods

### Multi-informant data sets and procedure

This research uses data collected as part of an ongoing longitudinal project between the Australian National University (ANU) and the Australian Capital Territory (ACT) Education and Training Directorate (ETD) (Reynolds et al., 2012).^1^ The project aimed to measure and enrich the health of school climates in the district in order to improve student and staff outcomes. The project involved all 86 public schools in the district, a city region with a population of ~367,752 (Australian Bureau of Statistics, 2014).

The present study also uses educational register data from 2,257 students' achievement scores on a robust, standardized, and nation-wide test, the Australian National Assessment Program—Literacy and Numeracy (NAPLAN). Every 2 years, all Australian students from Grade 3 up to Grade 9 sit NAPLAN tests. The current study sampled Grades 7–10 (high school attendees in the district) students' scores, which were provided by the education department.

Specifically, the following three data sets were merged to a single main data set.

1. Data from education district records. This included demographic information, such as levels of parental education, school level SES, and student achievement scores. Student achievement scores are a sample of students' results on 2014 NAPLAN tests.
2. Student survey responses. An online survey was administered to all Grade 7 and 9 students at all schools in the ACT during a 2-week period (during June 2014). Students provided their consent if they chose to participate. Then they completed the online survey (through Qualtrics software) in their classrooms with teachers' assistance. Parents' consent was waved by the relevant authority due to the low risk nature of the survey and students being able to provide own consent. Survey responses were on a Likert scale from 1 (“disagree strongly”) to 7 (“agree strongly”). Data sets 1 and 2 were merged to include students who both participated in the SCASIM-St survey, and completed NAPLAN tests. Accordingly, each student's survey response was matched with their NAPLAN scores and demographic information.
3. Staff responses from the SCASIM-Sf survey. Staff provided consent and completed an online survey during the survey period at a time convenient to them.

### Participants

The sample included 2,257 Grade 7 and 9 students and 760 staff from 17 public schools, 89% of all the 19 public high schools in the district.

#### Students

One thousand and one hundred fifteen male students (49.4%) and one thousand and one hundred forty-two female students took part in the survey (*M* = 13.3 years old, *SD* = 1.2). 51.5% were in Grade 7 and 48.5% were in Grade 9. 80.3% of the sample spoke English at home, compared to the overall Australian average of 82% (Australian Bureau of Statistics, 2014). 0.7–1% participants who did not indicate their their age, spoken language at home, or gender, were excluded from the main analysis. 65.3% of the students' parents had education levels below a university level. The survey response rate was between 23.7 and 79% (*M* = 61.47%). This percentage can be attributed to some students being absent, some deciding not to participate, and there being some technological issues with online participation. The response rate was included as a covariate in the statistical models to control for possible sampling issues, and was placed at the school level in the MLM.

#### Staff

The staff sample consisted of 497 females (68.6%) and 228 males (31.4%), which is representative of the female majority of educators in Australia (Australian Bureau of Statistics, 2014). The average age was 41.03 years old (*SD* = 11.5, range = 18–70). 15.2% were administrative staff and 84.3% were teaching staff. Administrative staff members were included because they play a role in setting and reflecting the climate of the schools. 4.6% participants did not report their gender and 7.5% did not report their age, so they were excluded from the main analysis.

#### Schools

Among the 17 schools, the average school size was 676.54 students (*SD* = 274.12, range = 205–1154). 58.82% were Kindergarten to Grade 10 schools and 41.18% were high schools containing Grades 7–10. An average of 25.42% of students in each school had a language background other than English (range = 13–65%, one school was a bilingual school, with 65% of students with a language background other than English). The SES of the schools was measured by the Index of Community Socio-Educational Advantage (ICSEA, described later in detail). On a possible scale from 500 to 1,300, ICSEA values for the schools ranged from 971.68 to 1177.91 (*M* = 1075.21, *SD* = 57.24).

### Measures

#### Student measures

##### Students' perceptions of school climate and level of school identification

School Climate and School Identification Measurement Scales-Student (SCASIM-St, Lee et al., 2017) with 38 items was used to measure school climate and school identification. The four subscales for school climate are academic emphasis (8 items, α = 0.929), staff-student relations (9 items, α = 0.964), student-student relations (7 items, α = 0.959), and shared values and approach (8 items, α = 0.927). The school identification factor consists of 6 items (α = 0.944). The subscales were highly reliable with the current data (αs > 0.7). The SCASIM-St has also shown criterion validity associated with academic achievement, attendance, aggressive behavior at school, and a well-being factor of depression (Lee et al., 2017).

##### Demographic variables

Students' age, gender, spoken language at home, and parents' level of education was collected by the survey or matched from education records.

##### Students' academic achievement

Grade 7 and 9 students' performance on NAPLAN tests was used to measure academic achievement in numeracy, reading and writing ability. Students' scores are standardized and range from 0 to 1,000 (Australian Curriculum Assessment Reporting Authority, 2014).

#### Staff measures

##### Staff perceptions of school climate and school identification

These were measured by a staff measure, the School Climate and School Identification Measurement Scales-staff (SCASIM-Sf, the scale's factor structure was validated in a supplementary analysis, and available as supplementary Material). It was used as a paired and mirrored scale of the student version, The confirmatory factor analysis (CFA) on the SCASIM-Sf^2^ revealed that the 36 items represent four sub-factors of school climate and school identification, as parallel with the student survey. The factors were reliable with the data: academic emphasis (8 items, α = 0.94), staff-student relations (9 items, α = 0.95), staff-staff relations (5 items, α = 0.94), shared values and approach (8 items, α = 0.94), and a correlated school identification factor (6 items, α = 0.95).

#### School level measures

##### School-wide SES schools'

SES levels were measured by the Index of Community Socio-Educational Advantage (ICSEA) and included as a covariate. ICSEA values are nationally standardized to reflect educational advantages and disadvantages at the school level, based on student family and school background variables such as parents' occupation and school location. Higher values indicate more advantages for the school students and the values are on a scale from 500 to 1,300 (median = 1,000, The Australian Curriculum, Assessment and Reporting Authority, 2014).

##### School size

School size was measured by the number of students enrolled in the school and was included as a covariate. This information was from the 2014 district school census.

### Analytical plan

The variance in students' achievement scores was analyzed at both the within (individual) level and between (school) level, due to substantial intra-class correlations (ICCs) and subsequent design effects above two^3^. As detailed in the following section, the results suggested that responses within schools were not independent (Hox, 2010). To handle this dependency, two-level multilevel Structural Equation Modeling (SEM) procedures were employed using MPlus version 7 (Muthén and Muthén, 1998–2015). Hierarchical models were tested to assess the impacts of student and staff perceptions of school climate and school identification on students' NAPLAN results. The impact of covariates on NAPLAN results was also examined in all models from the base model.

Variables on the first-level of the model (“within-level” or “individual level”) were students' grade, gender, parents' educational level, and students' perceptions of school climate and school identification. Staff members' school climate perception and school identification were also placed on the within-level. Staff ratings may well-serve as school-level variables, however the current data exhausted all the school-level variance once the school-level demographics were controlled for. Therefore, staff variables were modeled to further explain individual student-level variance. Specifically staff perceptions of school climate were averaged as school means and disaggregated on to individual student data. Thus, students from the same school had the same staff school climate mean scores in their data. This practice (disaggregating the school means to the individual level to explain the variance in individual students' scores) has been applied before in educational research to explain achievement (e.g., Thomas and Collier, 2002).

Second-level (“between-level” or “school level”) variables were covariates, including schools' ICSEA value and size, as well as the student response rate. School-level variables were entered as random effects (as they were expected to differ between schools) and individual-level variables were declared fixed (it was presumed that there would be no random differences in the relationships between the variables and NAPLAN results).

First, the “null models” (“Model 0”) were run to get the ICCs, which determined the proportion of variance accounted for by the clustering (Goldstein et al., 2002), and confirmed whether MLM procedures were required. Models were then built hierarchically, increasing in complexity and explanatory potential as more predictor variables were added.

Model 1 was then tested, adding covariate demographic variables at the student and school levels. These models operated as a baseline for models 2–7, to compute the increased proportion of explained variance (Δ*R*^2^) as other variables were added to the models. Student perceptions were then added in Model 2 to test the impact on academic achievement. Social identity mediation was then tested with two subsequent models, first by adding student school identification to Model 3 and then by modeling school identification as a mediator (Model 4).

As the next step, staff perceptions of school climate were added to test the impact) in Model 5 with all other variables controlled. Staff's school identification was added to Model 6, and then an interaction term was added to test if staff social identity significantly moderated the impact of staff school climate perception on student achievement (Model 7). Model 7 was the most complex model run, and is visually depicted in Figure 2.

Domain specificity was anticipated to occur (Marsh et al., 2005; Hinnant et al., 2009), wherein the nature and extent of the impact of the variables on NAPLAN results may have varied according to subject domain. Correspondingly, Models 0–7 were run for each of the three different dependent variables (numeracy, reading, and writing results)^4^.

## Results

### Descriptive statistics

Data screening showed that both the staff and student data sets were not normally distributed. The means, standard deviations, skew, kurtosis, and reliability statistics for the staff and student school climate sub-scales are reported in Tables 1, 2, respectively. For staff responses, all means were reasonably high on the 7-point Likert scale, with a small range (5.22–5.98), as were the student responses (4.01–5.02). The dependent variables (students' scores on numeracy, reading, and writing NAPLAN tests) were also not normally distributed. Out of a possible score of 1,000, students' overall total means were 565.43 for numeracy (*SD* = 83.38), 572.70 for reading (*SD* = 87.72), and 525.09 for writing (*SD* = 106.43). Therefore, non-normality was dealt with the MPlus MLR estimator (maximum likelihood estimation with robust standard errors) that are robust to non-normality (Muthén and Muthén, 1998–2015). The data missing rate was trivial with a maximum of 2.8–0.7% at average. Accordingly a multiple imputations method was employed using Mplus.

**Table 1:** Means, standard deviations, skewness and kurtosis scores, and cronbach's alpha for the scale scores in the student and staff samples.

**Sample**	**Sub-scale/Sub-factor**	***M***	***SD***	**Skew**	**Kurtosis**	**α**
Students	School identification	4.71	1.44	−0.45	−4.1	0.94
	Shared values and approach	4.62	1.31	−0.42	−0.27	0.93
	Staff-student relations	4.55	1.49	−0.34	−0.51	0.96
	Student-student relations	4.01	1.46	−0.12	−0.61	0.96
	Academic emphasis	5.02	1.32	−0.64	0.01	0.93
Staff	School identification	5.88	1.12	−1.17	1.14	0.95
	Shared values and approach	5.25	1.19	−0.78	0.31	0.94
	Staff-student relations	5.98	0.81	−1.21	2.23	0.95
	Academic emphasis	5.83	0.89	0.87	0.86	0.94
	Staff-staff relations	5.22	1.27	−0.80	0.25	0.94

*Student N = 2,257; Staff N = 760; α = Cronbach's alpha*.

**Table 2:** Correlations among study variables, means, and standard deviations.

**Variables**	**1**	**2**	**3**	**4**	**5**	**6**	**7**	**8**	**9**	**10**	**11**	**12**	**13**	**14**	**15**	**16**	**17**	**18**	**19**
1. Numeracy	—																		
2. Reading	0.77^**^	—																	
3. Writing	0.62^**^	0.66^**^	—																
4. SVA	0.06^**^	0.02	0.07^**^	—															
5. AcaEmp	0.02	0.94	0.12	0.79^**^	—														
6. StdRel	0.11^**^	0.04	0.07^**^	0.69^**^	0.61^**^	—													
7. SfdRel	0.03	−0.01	0.03	0.78^**^	0.81^**^	0.65^**^	—												
8. SchId	0.12^**^	0.07^**^	0.11^**^	0.78^**^	0.69^**^	0.64^**^	0.67^**^	—											
9. Grade	0.25^**^	0.17^**^	0.15^**^	−0.14^**^	−0.17^**^	−0.08^**^	−0.13^**^	−0.13^**^	—										
10. Gender^a^	−0.07^**^	0.04^**^	0.19^**^	−0.01	−0.01	−0.02	−0.03	−0.02	−0.02	—									
11. PrtUni^b^	0.26^**^	0.25^**^	0.20^**^	0.06^**^	0.05^*^	0.10^**^	0.05^*^	0.10^**^	0.01	−0.02	—								
12. ICSEA	0.33^**^	0.28^**^	0.21^**^	0.13^**^	0.05^*^	0.18^**^	0.02	0.17^**^	0.04	−0.03	0.33^**^	—							
13. School size	0.30^**^	0.25^**^	0.19^**^	0.14^**^	0.06^**^	0.17^**^	0.03	0.17^**^	0.02	0.00	0.25^**^	0.86^**^	—						
14. Res Rate	0.05^*^	0.036	0.039	0.02	−0.01	0.03	−0.02	0.02	−0.06^**^	0.03	0.10^**^	0.08^**^	0.09^**^	—					
15. SfSchId	0.13^**^	0.15^**^	0.12^**^	0.12^**^	0.11^**^	0.11^**^	0.08^**^	0.14^**^	0.02	−0.01	0.12^**^	0.30^**^	0.24^**^	0.25^**^	—				
16. SfSVA	0.21^**^	0.22^**^	0.18^**^	0.14^**^	0.11^**^	0.15^**^	0.07^**^	0.16^**^	0.01	−0.00	0.22^**^	0.51^**^	0.44^**^	0.45^**^	0.91^**^	—			
17. SfSfSdRl	0.09^**^	0.10^**^	0.09^**^	0.04^*^	0.06^**^	0.07^**^	0.03	0.07^**^	−0.03	0.01	0.09^**^	0.16^**^	0.16^**^	0.33^**^	0.85^**^	0.81^**^	—		
18. SfAcaEmp	0.16^**^	0.15^**^	0.14^**^	0.07^**^	0.06^**^	0.08^**^	0.02	0.11^**^	−0.59^**^	0.03	0.15^**^	0.37^**^	0.33^**^	0.41^**^	0.72^**^	0.80^**^	0.85^**^	—	
19. SfSfRel	0.06^**^	0.09^**^	0.08^**^	0.09^*^	0.10^**^	0.07^**^	0.07^**^	0.08^**^	−0.03	0.01	0.05^*^	0.05^*^	0.10^**^	0.45^**^	0.85^**^	0.82^**^	0.88^**^	0.74^**^	—
*Mean*	565.59	572.90	525.28	4.62	5.02	4.01	4.55	4.71	—	—	—	1075.21	676.54	61.47	5.89	5.28	5.97	5.83	5.24
*SD*	83.06	87.25	106.07	1.31	1.32	1.47	1.46	1.45	—	—	—	57.24	274.12	14.01	0.25	0.47	0.21	0.24	0.39

N = 2,257. SVA, shared values and approach; AcaEmp, academic emphasis; StdRel, student-student relations; SfdRel, staff-student relations; SchId, school identification; PrtUni, parental education; Res rate, students' response rate; SfSchId, staff school identification; Grade, School Grade; “Sf” prefix denotes staff perceptions of the school climate subfactors;

a Gender: male coded 0, female coded 1;

b *Parental education: below university degree coded 0, university degree or higher coded 1*.

* *p < 0.05*,

** *p < 0.01*.

After screening and cleaning the data, the staff data was merged with the student data set by disaggregating staff responses as school means. The final data set included students' demographic variables, students' ratings of school climate and school identification, staff ratings of school climate and school identification, school-level demographic variables and NAPLAN scores. This merged data set was then used for analyzing correlations (Table 2)^5^ and for the main multilevel SEM analysis.

### Multilevel SEM analysis

It was expected that students' perceptions of school climate would be positively related to students' NAPLAN results (H1) and that this relationship would occur through students' school identification (H2). It was also expected that staff perceptions of school climate would be positively related to student achievement (H3) and that staff's school identification would moderate this relationship (H4).

#### Multilevel modeling

The ICCs and design effects for the numeracy, reading, and writing models were high enough to require multilevel modeling (ICCs: numeracy: 0.08, reading: 0.05, writing: 0.04, all design effects >2, (Satorra and Muthen, 1995; Muthén and Muthén, 2009; Hox, 2010; Muthen and Muthen, 2007). The maximum likelihood parameter estimation with standard errors (MLR) was used because it is robust to non-normality, enabling the analysis of the substantially skewed and kurtosed data (Muthén and Muthén, 1998–2015). Tables 3–8 summarize the results of the hierarchical stepwise multilevel SEMs. Models 0–6 were run separately, with writing, reading, and numeracy scores as dependent variables.

**Table 3:** Multilevel SEM results for models 0–3 explaining NAPLAN numeracy.

	**Model 0**	**Model 1^d^**		**Model 2**		**Model 3**	
		***b***	**(s.e.)**	***b***	**(s.e.)**	***b***	**(s.e.)**
**LEVEL 1 PREDICTORS**							
Grade	—	19.73 ^**^	2.59	20.34 ^**^	2.44	18.86^**^	2.42
Gender^a^	—	−9.68^*^	4.06	−9.47^*^	4.01	−9.79^*^	3.98
Parental education^b^	—	29.37^**^	1.84	29.17^**^	1.90	28.37^**^	1.97
StSchClim	—	—	—	3.80^†^	2.04	−4.85	3.96
StSchId	—	—	—	—	—	9.00^**^	2.66
**LEVEL 2 PREDICTORS**							
ICSEA^c^	—	0.24^**^	0.07	0.24^**^	0.07	0.22^*^	0.09
School size	—	0.04	0.02	0.03^†^	0.02	0.04	0.02
Response rate	—	0.04	0.36	0.07	0.41	0.10	0.56
Level 2 Intercept	—	396.81		392.15^**^		366.92^**^	
**MODEL STATISTICS**							
Within-school variance	6051.73^**^	5553.19^**^		5541.12^**^		5494.58^**^	
Between-school variance	3393.02^**^	20.18		12.77		2.54	
χ^2^ (*df*)	—	—		93.18(16)^**^		161.62(22)^**^	
CFI	—	—		0.98		0.98	
RMSEA	—	—		0.05		0.05	
Within-school *R^2^*	—	0.08		0.09		0.09	
Total *R^2^*	—	0.41		0.41		0.42	

*N = 2,257*.

a Gender: 0 = male, female = 1;

b Parental education, 1 = university degree or higher, 0 = lower than university degree;

c ICSEA, Index of Community Socio-Educational Advantage;

d *Model 1 was a regression model not involving model fit statistics compared to the other SEMs; s.e, Standard Error; CFI, Comparative Fit Index; RMSEA, Root Mean Square Error of Approximation; StSchClim, Student perceptions of school climate; StSchId, Students' school identification. Dashes indicate that the variable was not entered in the model*.

† p < 0.10;

* p < 0.05;

** *p < 0.01(two-tailed tests)*.

**Table 4:** Multilevel SEM results for models 4–6 explaining NAPLAN numeracy.

	**Model 4**		**Model 5**		**Model 6**	
	***B***	**(s.e.)**	***b***	**(s.e.)**	***b***	**(s.e.)**
**LEVEL 1 PREDICTORS**						
Grade	20.62^**^	2.45	20.52^**^	2.41	20.88^**^	2.39
Gender^a^	−9.31^*^	4.03	−9.29^*^	4.12	−9.30^*^	4.1
Parental education^b^	28.44^**^	3.18	28.36^**^	1.68	28.26^**^	1.65
StSchClim	−6.89^†^	3.97	−6.88^†^	3.96	−6.97^†^	3.94
StSchId	10.03^*^	3.18	9.91^*^	3.13	10.21^*^	3.13
StSchClimSchID^X^	1.08^**^	0.03	1.09^**^	0.03	1.09^**^	0.03
Indirect path via StSchId^Y^	10.90^*^	3.45	10.78^*^	3.40	11.10^*^	3.40
SfSchClim	—	—	7.57^*^	3.63	29.67	18.06
SfSchId	—	—	—	—	−38.94	27.51
**LEVEL 2 PREDICTORS**						
ICSEA^c^	0.22^*^	0.09	0.21^**^	0.05	0.22^**^	0.06
School size	0.04	0.02	0.04^**^	0.01	0.03^*^	0.01
Response rate	0.09	0.61	0.02	0.18	−0.06	0.21
Level 2 Intercept	343.27^**^	344.97^**^	569.95^*^
**MODEL STATISTICS**						
Within-school variance	5483.37^**^	5480.91^**^	5464.71^**^			
Between-school variance	7.98	3.00	1.96			
χ^2^ (*df*)	166.18 (22)^**^	1892.11 (78)^**^	217.76 (70)^**^			
CFI	0.98	0.95	0.91			
RMSEA	0.06	0.03	0.03			
Within-school *R^2^*	0.09	0.09	0.10			
Total *R^2^*	0.42	0.42	0.42			

*N = 2,257*.

a Gender: 0 = male, female = 1;

b Parental education, 1 = university degree or higher, 0 = lower than university degree;

c ICSEA, Index of Community Socio-Educational Advantage;

*^d^Model 1 was a regression model not involving model fit statistics compared to the other SEMs; s.e, Standard Error; CFI, Comparative Fit Index; RMSEA, Root Mean Square Error of Approximation; StSchClim, Student perceptions of school climate; StSchId, Students' school identification. Dashes indicate that the variable was not entered in the model*.

† *p < 0.10*;

* *p < 0.05*;

** *p < 0.01(two-tailed tests)*.

**Table 5:** Multilevel SEM results for models 0–3 explaining NAPLAN reading.

	**Model 0**	**Model 1^d^**		**Model 2**		**Model 3**	
		***b***	**(s.e.)**	***b***	**(s.e.)**	***b***	**(s.e.)**
**LEVEL 1 PREDICTORS**							
Grade	—	13.69^**^	2.55	13.58^**^	2.65	13.80^**^	2.61
Gender^a^	—	9.45^†^	5.31	9.43^†^	5.27	9.59^†^	5.29
Parental education^b^	—	33.52^**^	2.60	33.56^**^	2.59	33.11^**^	2.60
StSchClim	—	—	—	−0.66	2.23	−8.43	5.36
StSchId	—	—	—	—	—	7.15^†^	4.22
**LEVEL 2 PREDICTORS**							
ICSEA^c^	—	0.25^*^	0.10	0.25^*^	0.11	0.24^*^	0.11
School size	—	0.02	0.02	0.02	0.02	0.02	0.02
Response rate	—	−0.14	0.36	−0.15	0.36	−0.14	0.37
Level 2 Intercept	—	440.08^*^		440.896^**^		405.77^**^	
**MODEL STATISTICS**							
Within-school variance	6923.45^**^	6561.93^**^		6560.365^**^		6529.98^**^	
Between-school variance	3719.04^**^	66.38		69.139		67.29	
χ^2^ (*df*)	—	—		85.805(16)^**^		126.43(22)^**^	
CFI	—	—		0.98		0.98	
RMSEA	—	—		0.04		0.05	
Within-school *R^2^*	—	0.05		0.05		0.06	
Total *R^2^*	—	0.38		0.38		0.39	

*N = 2,257*.

a *Gender: 0 = male, female = 1*;

b *Parental education, 1 = university degree or higher, 0 = lower than university degree*;

c *ICSEA, Index of Community Socio-Educational Advantage*;

d *Model 1 was a regression model not involving model fit statistics compared to the other SEMs; s.e, Standard Error; CFI, Comparative Fit Index; RMSEA, Root Mean Square Error of Approximation; StSchClim, Student perceptions of school climate; StSchId, Students' school identification. Dashes indicate that the variable was not entered in the model*.

† *p < 0.10*;

* *p < 0.05*;

** *p < 0.01(two-tailed tests)*.

**Table 6:** Multilevel SEM results for models 4–6 explaining NAPLAN reading.

	**Model 4**		**Model 5**		**Model 6**	
	***b***	**(s.e.)**	***b***	**(s.e.)**	***b***	**(s.e.)**
**LEVEL 1 PREDICTORS**						
Grade	13.80^**^	2.61	13.89^**^	2.55	13.89^**^	2.55
Gender^a^	9.59^†^	5.29	9.80^†^	5.20	9.80^†^	5.21
Parental education^b^	33.11^**^	2.60	33.10^**^	2.46	33.02^**^	2.52
StSchClim	−8.43	5.36	−8.14	5.3	−8.05	5.32
StSchId	7.15^†^	4.22	7.07^†^	4.20	7.06^†^	4.19
StSchClimSchID^X^	1.1^**^	0.03	1.1^**^	0.03	1.1^**^	0.03
Indirect path via StSchId^Y^	7.87^†^	4.69	7.78^†^	4.67	7.77^†^	4.65
SfSchClim	—	—	16.80^*^	5.34	34.17	23.74
SfSchId	—	—	—	—	−28.38	35.50
**LEVEL 2 PREDICTORS**						
ICSEA^c^	0.24^*^	0.11	0.20^*^	0.07	0.20^*^	0.08
School size	0.02	0.02	0.03^*^	0.01	0.02^*^	0.01
Response rate	0.14	0.37	−0.17	0.20	−0.26	0.24
Level 2 Intercept	405.76^**^		407.29^**^		574.73^*^	
**MODEL STATISTICS**						
Within-school variance	6529.99^**^		6543.17^**^		6532.59^**^	
Between-school variance	67.25		3.00		1.96	
χ^2^ (*df*)	126.44(22)^**^		145.68(59)^**^		222.57(70)^**^	
CFI	0.98		0.95		0.91	
RMSEA	0.05		0.03		0.03	
Within-school *R^2^*	0.06		0.06		0.06	
Total *R^2^*	0.39		0.39		0.39	

*N = 2,257*.

a *Gender: 0 = male, female = 1*;

b *Parental education, 1 = university degree or higher, 0 = lower than university degree*;

c *ICSEA, Index of Community Socio-Educational Advantage; s.e, Standard Error; CFI, Comparative Fit Index; RMSEA, Root Mean Square Error of Approximation; StSchClim, Student perceptions of school climate; StSchId, Students' school identification. x, the path from school climate to school identification; y, the indirect path from student school climate to achievement score via school identification*.

† *p < 0.10*;

* *p < 0.05*;

** *p < 0.01(two-tailed tests)*.

**Table 7:** Multilevel SEM results for models 0–3 explaining NAPLAN writing.

	**Model 0**	**Model 1**^**d**^		**Model 2**		**Model 3**	
		***b***	**(s.e.)**	***b***	**(s.e.)**	***b***	**(s.e.)**
**LEVEL 1 PREDICTORS**							
Grade	—	15.85^**^	1.46	16.82^**^	1.41	17.21^**^	1.38
Gender^a^	—	42.11^**^	6.64	42.34^**^	6.57	42.52^**^	6.62
Parental education^b^	—	31.13^**^	4.02	30.87^**^	4.06	30.01^**^	3.82
StSchClim	—	—	—	4.80^*^	2.39	−6.769	6.21
StSchId	—	—	—	—	—	10.92^*^	4.86
**LEVEL 2 PREDICTORS**							
ICSEA^c^	—	0.24^†^	0.15	0.23^†^	0.14	0.22	0.14
School size	—	0.01	0.03	0.1	0.03	0.01	0.03
Response rate	—	−0.23	0.43	−0.20	0.42	−0.19	0.43
Level 2 Intercept	—	359.48^**^		352.01^**^		298.11 ^**^	
**MODEL RESULTS**							
Within-school variance	10503.58^**^	9800.87^**^		9779.32^**^		9706.62^**^	
Between-school variance	5515.43^**^	165.99		147.61		144.63	
χ^2^ (*df*)	—	0.01(0)^**^		65.25 (14)^**^		2159.15(20)^**^	
CFI	—	—		0.99		0.99	
RMSEA	—	—		0.04		0.05	
Within-school *R^2^*	—	0.07		0.07		0.08	
Total *R^2^*	—	0.39		0.39		0.39	

*N = 2,257*.

a *Gender: 0 = male, female = 1*;

b *Parental education, 1 = university degree or higher, 0 = lower than university degree*;

c *ICSEA, Index of Community Socio-Educational Advantage; s.e, Standard Error; CFI, Comparative Fit Index; RMSEA, Root Mean Square Error of Approximation; StSchClim, Student perceptions of school climate; StSchId, Students' school identification. x, the path from school climate to school identification; y, the indirect path from student school climate to achievement score via school identification*.

† *p < 0.10*;

* *p < 0.05*;

** *p < 0.01(two-tailed tests)*.

**Table 8:** Multilevel SEM results for models 4–6 explaining NAPLAN writing.

	**Model 4**		**Model 5**		**Model 6**	
	***b***	**(s.e.)**	***b***	**(s.e.)**	***b***	**(s.e.)**
**LEVEL 1 PREDICTORS**						
Grade	17.22^**^	1.40	17.22^**^	1.38	17.48^**^	1.41
Gender^a^	42.64^**^	6.62	42.64^**^	6.62	42.69^**^	6.62
Parental education^b^	30.07^**^	3.90	30.07^**^	3.90	30.13^**^	3.88
StSchClim	1.09^**^	0.03	−6.90	6.17	−7.00	6.15
StSchId	10.89^*^	4.87	10.89^*^	4.87	11.16^*^	4.86
StSchClimSchID^X^	1.08^**^	0.03	1.08^**^	0.03	1.08^**^	0.03
Indirect path via StSchId^y^	11.83^*^	5.37	11.83^*^	5.37	11.87^*^	5.36
SfSchClim	—	—	21.21^*^	8.55	41.68^†^	22.59
SfSchId	—	—	—	—	−37.83	36.17
**LEVEL 2 PREDICTORS**						
ICSEA^c^	0.16	0.11	0.16	0.11	0.17	0.12
School size	0.02	0.02	0.02	0.02	0.02	0.02
Response rate	−0.31	0.40	−0.31	0.40	−0.35	0.38
Level 2 Intercept	300.12^**^		300.12^**^		362.25^*^	
**MODEL RESULTS**						
Within-school variance	9703.72^**^		9703.72^**^		9690.72^**^	
Between-school variance	82.72		82.72		64.03	
χ^2^ (*df*)	1882.90(78)^**^		141.32(58)^**^		1779.20(91)^**^	
CFI	0.95		0.95		0.91	
RMSEA	0.03		0.03		0.03	
Within-school *R^2^*	0.08		0.08		0.08	
Total *R^2^*	0.39		0.39		0.40	

*N = 2,257*.

a *Gender: 0 = male, female = 1*;

b *Parental education, 1 = university degree or higher, 0 = lower than university degree*;

c *ICSEA, Index of Community Socio-Educational Advantage; s.e, Standard Error; CFI, Comparative Fit Index; RMSEA, Root Mean Square Error of Approximation; StSchClim, Student perceptions of school climate; StSchId, Students' school identification. x, the path from school climate to school identification; y, the indirect path from student school climate to achievement score via school identification*.

† *p < 0.10*;

* *p < 0.05*;

** *p < 0.01(two-tailed tests)*.

#### Demographic covariates

The demographic covariate-only model (Model 1) showed that 8.4, 5.2, and 6.7% of variance in numeracy, writing, and reading performance, respectively, was explained by the three covariates of grade, gender, and parental education (Tables 3, 5, 7). However, the school-level variances (20.18~165.99, *p* = 0.19~0.76) were completely explained by the three school-level covariates of SES, school size, and response rate, before including staff perception variables on school climate or identification. These results forced analyzing the staff variables at the student level, as the exhausted variance at the school-level meant no additional explanatory variable could be added at the school level of the model.

When all other variables were included (Model 6), the impact of most covariates on achievement persisted. For example, NAPLAN scores were significantly higher for students in higher grades (writing *b* = 17.48, *p* < 0.01; reading *b* = 13.89; numeracy *b* = 20.88) and students who had parents with higher educational levels (writing *b* = 30.13, *p* < 0.01; reading *b* = 33.02; numeracy *b* = 28.26). Boys performed better than girls on numeracy tests (*b* = −9.30, *p* < 0.05) and girls performed better on literacy tests, particularly writing tests (reading *b* = 9.59, *p* < 0.10; writing *b* = 42.69, *p* < 0.01).

School-level variables showed mixed effects. Response rate did not significantly predict student achievement at the school level in any domain. However, larger schools (reading: *b* = 0.02, *p* < 0.05; numeracy: *b* = 0.03, *p* < 0.05) and schools with higher SES had significantly higher achievement at the school level in numeracy *(b* = 0.22, *p* < 0.01) and reading *(b* = 0.20, *p* < 0.05). There was no effects of school covariates on writing achievement at the school level, when all the variables including school climate perception of both student and staff groups and student school identification.

#### Social identity mediation: students' school climate perception impacted on numeracy, writing, and reading through their school identification

As shown in the Model 4 results in Table 2, students' school identification (*b* = 10.03, *p* < 0.05) completely mediated the impact of their school climate perception (indirect effect, *b* = 10.90, *p* < 0.05) upon Numeracy. For Writing (Model 4 in Table 3), partial mediation was observed with the significant impact of students' school identification (*b* = 10.89, *p* < 0.05) as well as their school climate perception (*b* = 1.09, *p* < 0.05; indirect effect, *b* = 11.83, *p* < 0.05). Yet, only marginally significant mediation effects were examined for Reading (Model 4 in Table 4) with students' school identification (*b* = 7.15, *p* < 0.10) and their perceptions of school climate (indirect effect, *b* = 7.87, *p* < 0.10). In all models, students' perception of school climate impacted their school identification (*b* = 1.08–1.1, *p* < 0.01). All these results were so when all the individual and school level covariates were taken into account.

#### Staff school climate perception impacted on student's numeracy, writing, and reading achievement

As presented in Model 5 in Tables 3–5, staff perceptions of school climate were significant predictors of students' academic achievement (writing *b* = 21.21; reading *b* = 16.80; numeracy *b* = 7.57, all *p* < 0.05). However, staff's school identification was not a significant predictor of students' academic achievement (Model 6, Tables 3–5). Because staff school identification was not found to be a significant predictor of students' numeracy, reading, or writing results, the seventh proposed model (suggesting staff's school identification as a moderator) could not be investigated.

Overall, Model 5 was the most complicated model run, as it had better model fit than Model 6 and others. Model 5 included demographic covariates, students' perceptions of school climate (with students' school identification modeled as a mediator) and staff perceptions of school climate to explain NAPLAN results. This model is visually depicted in Figure 2 for numeracy achievement scores. The fifth model uniquely explained 8, 6, and 9% of variance at the student level in students' writing, reading, and numeracy scores, respectively. In total, 39% for both variances in writing and reading, and 42% of variance in numeracy scores were explained by Model 5 with the student and school level variables.

## Discussion

This study used a multilevel framework to examine the influence of individual (student and staff) factors and school level factors on students' academic achievement. Three out of the four hypotheses were supported. Positive student and staff perceptions of school climate positively and significantly impacted students' NAPLAN results, as expected. Students' school identification mediated the impact of their perception of school climate on their performance in two learning domains. However, staff's school identification did not moderate the impact of staff's perceptions on student achievement.

### Academic achievement explained by student- and school-level variables

Students' individual factors (gender, grade, and education level of their parents) and school factors (school size and school SES) significantly impacted students' academic achievement. Collectively, these factors accounted for 8.4, 5.2, and 6.7% of within-school variance in students' numeracy, writing, and reading performance respectively and ~40% of the whole variance in the achievement scores. As expected, consistent with the literature, boys tended to score better on numeracy and girls tended to score better on literacy (Halpern and LaMay, 2000; Marsh et al., 2005; Hinnant et al., 2009). The results also showed that the three most significant demographic predictors of student achievement were school SES, parental education and grade, replicating well-confirmed findings (Davis-Kean, 2005; Perry and McConney, 2010; Reynolds et al., 2017). However, student and staff perceptions of school climate also emerged as significant predictors in all three learning domains.

In line with the first hypothesis, the more positively students perceived school climate, the better their achievement scores were in the numeracy and writing domains. These results were evident even after known covariates of student achievement (gender, SES, and parental education) were controlled. Using a more complex model and a national standardized measure of achievement, this relationship between student school climate perception and achievement is largely consistent with the literature (Brookover et al., 1978; Sweetland and Hoy, 2000; Tschannen-Moran et al., 2006; Brand et al., 2008). School climate perception did not significantly impact reading performance, which reflects previous research demonstrating that the reading domain is less affected by school climate (Ma and Klinger, 2000; Reynolds et al., 2017).

The results showed substantial support for the second hypothesis, such that students' positive school climate perception predicted stronger school identification among students in all the three models of reading, writing, and numeracy, which in turn, predicted higher achievement scores in numeracy and writing. In other words, students' perceptions of school climate psychologically flowed through school identification to influence students' numeracy and writing scores (the indirect mediation effect was only marginally significant for reading performance). The current results demonstrate the impact of school climate may only operate indirectly, as a function of students' identification with the school. Students merely perceiving the school climate as positive might not be sufficient to trigger the influence of school climate on their achievement. Rather, school identification is a vital psychological mechanism to activate the influence of school climate on students' numeracy and writing performance.

The only other study to have directly tested the viability of school identification as a mechanism underpinning the climate-achievement link was conducted by Reynolds et al. (2017), using a much smaller sample (340 students in 2 schools). The present study replicated their findings that school identification mediated the impact of school climate on achievement in numeracy and writing, yet, with a much larger sample and MLM procedures. The findings are also consistent with Bizumic et al. (2009) and Turner et al. (2014), who provide evidence that school identification mediates the impact of school climate on non-academic outcomes, such as well-being and bullying behavior.

There are some important caveats on this interpretation. First, most students in this sample identified relatively strongly with their school (*M* = 4.71 on a 7-point Likert scale) so this mediation relationship may be generalizable to school populations in which students moderately to highly identify with the school. As noted, an effect of domain specificity was also apparent, so the mediation results should only be interpreted as applying to specific domains of students' numeracy and writing achievement.

### Academic achievement explained by staff variables

There was mixed support for the hypotheses for staff. Specifically, the results showed staff's perceptions of school climate significantly predicted students' academic achievement, confirming the third hypothesis. This finding is consistent with the literature (e.g., Johnson and Stevens, 2006; Brand et al., 2008; MacNeil et al., 2009; Yang, 2014). Given that student perceptions of school climate were controlled, the current research also gives more confidence in this school climate-achievement relationship from the staff perspective.

The fourth hypothesis was not supported. Staff's social identification did not significantly relate to, or moderate, the relationship between staff perceptions of school climate and students' academic achievement. It was expected that stronger identification would be associated with staff spending more time and effort on achieving the school's vision and norms, leading to better academic outcomes for students. Methodological limitations may have contributed to these non-significant findings. There may not have been enough statistical power for the multilevel model to detect a real effect. Tabachnick and Fidell (2014) advise that adequate power is generated when “sample sizes at the first level are not too small and the number of groups is 20 or larger” (p. 793). In this case, there were less than 20 groups (17 schools). The nature of the sample and variables may have also precluded a significant finding. The sample was quite homogenous and there was little variability in staff responses to school identification (*M* = 5.88 on a 1–7 Likert scale, *SD* = 1.12). Future research could overcome these limitations by including more schools and more diversity in respondents' levels of school identification.

In light of these methodological shortcomings, it is premature to extract theoretical meaning from the non-significant result for H4. However, it is plausible that there were other factors affecting staff members' job performance (measured by students' NAPLAN results) that were not included in the model, such as salary, leadership, training, and administrative support. Moreover, the model may not have captured the right *type* of identification. Staff could identify with other levels of identity that may affect their job performance (and hence, students' academic achievement). For example, staff's identification with the profession or teaching discipline (more broadly) or the classroom unit (more narrowly), may have impacted students' academic achievement. Clearly, this is fertile ground for further research.

### Implications for theory and research

This research has contributed to social-psychological and educational research concerning school climate and highlighted the importance of psychological factors for students' academic success. It replicated established findings that student and staff perceptions of school climate impact student achievement, and extended the research further by proposing school identification as an explanatory psychological mechanism for students. Various studies have explored the link between staff perceptions of school climate and student achievement, but none have controlled for student perceptions. Hence, for the first time in a single statistical model, the present study revealed the unique contribution of staff perceptions in explaining in student achievement. The study also contributed to the social identity body of work. The finding that school identification mediated the student-climate-achievement link is a marked contribution since schools are a relatively novel context to apply the theory (Reynolds and Branscombe, 2015).

The use of MLM procedures, national standardized academic achievement tests, a large sample size and the inclusion of covariates increased the reliability and validity of these findings. Importantly, no other study has used MLM procedures and national standardized academic achievement tests to explore the climate-achievement link. These findings are also strengthened by the multi-informant design of the study. As the introduction revealed, studies integrating multiple school climate perspectives are relatively rare in the school climate field (Thapa et al., 2013; Wang and Degol, 2015). Using multiple informants is considered “best practice” when measuring educational and psychological constructs (Konold and Cornell, 2015). Hence, this study has enriched the school climate field by including student and staff perspectives, answering research calls for measuring school climate from different perspectives (Thapa et al., 2013; Liu et al., 2014).

### Implications for designing school initiatives

By disentangling school-level factors from the student level factors affecting student achievement and illuminating core psychological processes within schools, the present study has also uncovered potential targets for intervention. Rather than standardized reforms that are insensitive to psychological elements of school functioning (e.g., economic incentives for teachers, increasing school resources), initiatives informed by this analysis could be more innovative by engaging with the psychological intricacies of school processes.

The following example initiatives are proposed as efficient strategies to affect change, since top-down change to the system level can capture more members than if every individual group member were to receive an individual intervention. It is presumably easier to change the health of the school climate and school members' school identification than to influence other factors, such as the SES of a school and other non-school factors that are beyond schools' control (Heck, 2000; Hoy et al., 2002). Two areas for targeted intervention are proposed; school climate perception and school identification.

#### Initiatives facilitating school climate and the perceptions by staff and students

Since school climate is malleable (Wang and Degol, 2015), interventions could modify and improve school members' perceptions of school climate in order to impact student achievement. For example, the Comer School Development Program (Cook et al., 2000) is an initiative that seeks to improve interpersonal relations and build shared academic and social goals among school members. After 2 years of the program's implementation, teachers' and students' ratings of schools' academic climate improved, as did students' results on mathematics and reading tests compared to controls. Another example is the Child Development Project, a school-wide intervention that seeks to foster healthy interpersonal relations (collaboration among and between staff, students, and parents) and a sense of common purpose (two sub-factors incidentally measured by the SCASIM). Results have shown that students who received this intervention felt more connected to the school and had significantly higher levels of academic achievement (measured by GPA and achievement test scores). The outcomes of the Child Development Project take on new importance when considering the mediation effect found in the present study. Hence, by strengthening school connectedness (identification) and increasing positive perceptions of school climate, the Child Development Project achieved two outcomes that this study has found to be critically related to student achievement.

#### Fostering school identification

Because students' psychological identification with a positive school climate emerged as a powerful variable influencing students' academic performance, interventions could foster and support students' feeling of closeness to the school. Turner et al. (2014) provide some guidance to this end, as the authors advocated for the implementation of the ASPIRe model^6^ (Haslam et al., 2003). The ASPIRe model operationalizes the core aspects of the social identity approach into a four-phase sequence of group tasks, which seek to foster increased organizational identification (school identification). In light of current results, activities which emphasize a shared mission of the school and remove barriers to psychological school membership might have positive implications for students' academic achievement.

### Limitations and future directions

First and foremost, this study would have benefitted from the inclusion of data from additional schools. Moreover, analyzing staff perceptions for the school level achievement would have made more statistical and theoretical sense, if staff perception ratings had not to be aggregated as means by school in the current study. However, this design was not possible as there was not enough variance left to explain at the school level, after school level variables such as school SES (ICSEA) and school size were accounted for. This situation may be explained by the fact that schools in the participating district are fairly homogenous in terms of student achievement due to the regional SES characteristics. This is in contrast to American schools (where much of the research has taken place), which are more diverse and for which school-level analysis was available (e.g., Brand et al., 2008). The inclusion of additional schools would have increased the power of the statistical model and may have enabled the analysis of staff perceptions on the school level. Hence, future studies should employ data from a larger number of schools to cross-validate the current findings.

Another statistical issue with the present study concerns high correlations between staff variables. The supplementary CFA analysis revealed staff school identification and the latent school climate factor were highly correlated (Online material A: *r* = 0.76). This already high correlation may have been further inflated when the staff data were disaggregated to the student data, as the correlation between shared values and approach and school identification increased from *r* = 0.71 in the CFA to *r* = 0.91 in the present study. Multicollinearity is a problem because multicollinear variables inflate error terms and weaken the analysis (Tabachnick and Fidell, 2014).

Similar to most of the school climate research, this study was neither longitudinal nor experimental. This is a problem for the research because causal inferences are not possible (Wang and Holcombe, 2010). Future studies examining causal relationships with interventions or a longitudinal design are clearly warranted (Brand et al., 2003). For example, differences in academic achievement could be measured after students receive an intervention that increases their school identification. An idea for investigating the climate-achievement link with a longitudinal design was put forward by Johnson and Stevens (2006, p. 119); “rather than relying on student achievement at one point in time, growth in student achievement could be used as an outcome construct.” This would be possible under the larger longitudinal project from which the current data set originated. For example, differences in the same cohort's level of academic achievement could be analyzed (the difference between NAPLAN data at time 1 [Grade 7] and time 2 [Grade 9]). A longitudinal design would also account for the fact that school climate perception is not static (Wang and Degol, 2015). It potentially changes and evolves during different points in the school year (for example, proximity to holiday periods or exam periods) and corresponding with different events at the school (for example, changing administration or exposure to a new initiative, Johnson and Stevens, 2006). Hence, longitudinal designs should be adopted in future research, as they would account for the impermanency of school climate perception (Wang et al., 2010).

Future studies should control for other known critical predictors of achievement. Even though many critical variables were included in this analysis, the most complicated models explained ~40% of the whole variance and only 7.6, 5.6, and 9.7% of variance at the student level in writing, reading, and numeracy scores, respectively. This reflects that teaching and learning is a complex process and numerous factors affect students' academic achievement. Future studies could include more covariates that have been known to influence academic achievement, such as students' individual SES, parental involvement, leadership, teacher credentials, students' IQ, students' motivation and attendance (Keith and Cool, 1992; Ma and Klinger, 2000; Perry and McConney, 2010). Students learning disabilities and attribution styles may also be important considerations as they can affect the student-teacher relationship (Pasta et al., 2013). In a comprehensive meta-analysis as a synthesis of more than 800 studies (over 50,000 studies) relating to academic achievement, Hattie (2009) found that among the most significant factors are feedback, metacognitive strategies and reciprocal teaching. Future studies might find that the additive role of such variables changes the strength of the impact of school climate and school identification on academic achievement, because school climate and school identification may also have significant predictors and determinants.

A strength of the present study was the inclusion of school climate as a latent construct in the models. It would also be interesting for future studies to test the impact of discrete sub-factors of school climate. Testing their respective roles on achievement may expose more precise areas for improvement (e.g., increasing academic emphasis for achievement). This means that interventions could be crafted to pinpoint those factors more directly (Wang and Degol, 2015). As noted previously, other types of social identification could also be tested, in order to further test the theoretical model. For example, classroom identification and peer-group identification could be tested for students, and workgroup identification and professional identification could be tested for staff.

## Conclusion

The present study aimed to deepen our understanding of the contributions of student and staff perceptions of school climate to student achievement. The findings have consolidated the importance of school climate and school identification for student achievement. The present study also aimed to uncover the psychological mechanisms underlying the climate-achievement link. This aim was partly achieved, as students' school identification emerged as a mediator in two out of three learning domains. This has illuminated potential targets for interventions and fertile ground for future research. Furthermore, through the use of multilevel modeling and measurement of multiple perspectives of school climate, the study addressed important methodological concerns identified in the literature. Overall, this study provided empirical support demonstrating that school climate and social identification are core variables that have the power to augment student achievement.

## Author contributions

EL and KR: contributed to the conception and design of the work; the acquisition, analysis, and interpretation of data for the work; and revising the work. SM: contributed to the conception and design of the work; the analysis and interpretation of data for the work; and drafting the work. ES: contributed to the conception and design of the work. DB: contributed to the conception and design of the work; and the acquisition of data for the work.

### Conflict of interest statement

The authors declare that the research was conducted in the absence of any commercial or financial relationships that could be construed as a potential conflict of interest.

## Footnotes

^1^The research project was approved by the research ethics committee at ANU (No. 2009/293).

^2^A series of CFA were conducted for the SCASIM-Sf used in the present study (see Supplementary Material A for a full analysis). Five plausible models were competitively tested in order to identify factor structures underlying and causing the 36 item responses. The most parsimonious model was selected, based on conventional model fit information and theoretical considerations. The final model for the SCASIM-Sf mirrored the SCASIM-St scale, and showed good model fit (χ^2^ = 2907.226 [p < .05], *df* = 580, CFI = 0.949, TLI = 0.945, SRMR = 0.051, RMSEA = 0.046).

^3^Formula for design effect (Satorra and Muthen, 1995; Muthén and Muthén, 2009): $DEFF=\;\frac{VC}{VSRS}=1\;\left(s-1\right)\rho$ VC = correct variance under cluster sampling; VSRS = variance assuming simple random sampling; s = common cluster size and ρ = intra-class correlation.

^4^**MULTILEVEL MODELING EQUATIONS** The following structural equations describe Model 7 (with numeracy as a dependent variable). This is an example of the last step of the hierarchical SEMs: **Level 1: student level (‘within school level’)**

(1) $$\begin{array}{l} Y{\left(Numeracy\;Score\right)}_{ij}=\;\beta_{0j}+\;\beta_{1j}Grade_{ij}+\;\beta_{2j}Gender_{ij}\\ \;+\;\beta_{3j}Parental\;Education_{ij}+\;\beta_{4j}Student\;School\;Climate_{ij}\\ \;+\;\beta_{5j}Student\;School\;Identification_{ij}+\;\beta_{6j}Staff\;School\;Climate_{ij}\\ \;+\;\beta_{7j}Staff\;School\;Identification_{ij}\\ \;+\;\beta_{8j}Staff\;School\;Climate\;X\;Staff\;School\;Identification_{ij}\;+\;e_{ij} \end{array}$$

(2) $$\begin{array}{l} Mediator\;{\left(Student\;School\;Identification\right)}_{ij}\\ \;=\;\beta M_{0j}+\;\beta M_{1j}Student\;School\;Climate_{ij} \end{array}$$

In the equations, the subscript *j* denotes the schools (*j* = 1, 2, 3 …17) and *i* is for individual students (*i* = *1, 2, 3….n)* within the school. βs are regression coefficients. β_0*j*_ represents the school intercept (latent factor) for the student's school which is estimated at the school level by Equation (3) in the following. β_1*j*_ is the regression coefficient of grade that represents the predicted increase in achievement scores by one unit in grade. β_8*j*_ is the regression coefficients of the interaction term of staff school climate perception and staff school identification. *e*_*ij*_ is an estimated error at the student level, i.e., random error of deviation in the student score that is unexplained by the equation. Therefore, an individual student's NAPLAN score, *Y*(*Numeracy Score*)_*ij*_ is the sum of the school intercept, β_0*j*_, for that student's school, all the serial predictor variables effect, and the individual level error, *e*_*ij*_. The mediator variable of students' school identification was also regressed on their school climate perception at the student level. **Level 2: school level (“between school level”)**

(3) $$\beta_{0j}=\;\gamma_{00}\;+\;\gamma_{01}SES_{j}+\;\gamma_{01}School\;Size_{j}+\;\gamma_{01}Response\;Rate_{j}+\;u_{0j}$$

In equation 3, γ denotes regression coefficients at the school level. An intercept for a school, β_0*j*_, is predicted by the average intercept over groups when all predictors are zero, γ_00_ (a fixed effect); regression coefficient of socio-economic status γ_01_, of school size γ_02_, of response rate γ_03_, and finally the school level error, *u*_0*j*_ (a random effect: deviation from average intercept for group j). **Multilevel Mode** If we integrate Model 7 into two equations by substituting Equation (3) into Equation (1), an individual student score would be estimated by the following Equation (4). In summary, a reading score of a student is the sum of the effects of school level predictors, student level predictors, and random errors (unexplained deviances) at both school and school level.

(4) $$\begin{array}{l} Y{\left(Numeracy\;Score\right)}_{ij}=\;\gamma_{00}\;+\;\gamma_{01}SES_{j}+\;\gamma_{01}School\;Size_{j}+\;\gamma_{01}Response\;Rate_{j}\\ \;+\;\beta_{1j}Grade_{ij}+\;\beta_{2j}Gender_{ij}+\;\beta_{3j}Parental\;Education_{ij}\\ \;+\;\beta_{4j}Student\;School\;Climate_{ij}+\;\beta_{5j}Student\;School\;Identification_{ij}\\ \;+\;\beta_{6j}Staff\;School\;Climate_{ij}+\;\beta_{7j}Staff\;School\;Identification_{ij}\\ \;+\;\beta_{8j}Staff\;School\;Climate\;X\;Staff\;School\;Identification_{ij}+\;u_{0j}\;+\;e_{ij} \end{array}$$

^5^As expected large intercorrelations between the four sub-factors of staff school climate perceptions (*r* > 0.5) were observed. These correlations suggested a latent general factor of school climate and they were analyzed as a measurement model (CFA) in the main SEM. One instance of multicollinearity was detected, between staff's perceptions of shared values and approach and staff's school identification (*r* = 0.91 > 0.9; Tabachnick and Fidell, 2014). Shared values and approach (‘SVA’) was a sub-factor of school climate, and analyzed in the factor structure of the higher order general school climate construct. Therefore, the multicollinearity between SVA and school identification did not directly affect the estimation of the parameters in the model. This multicollinearity issue may have arisen due to the disaggregation of staff data to the student data, which exaggerated the correlations that were identified in the CFA (supplementary analysis, Supplementary Material).

^6^ASPIRe is an acronym for Actualizing Social and Personal Identity Resources model.
