# Supplementary material for: The Impact of School Climate and School Identification on Academic Achievement: Multilevel Modeling with Student and Teacher Data
Source: Front Psychol. 2017 Dec 5;8:2069. doi: 10.3389/fpsyg.2017.02069 (PMC5723344; doi:10.3389/fpsyg.2017.02069)
Supplement: Supplementary file 1 [file Table1.DOCX]

Supplementary Material

The Impacts of on Academic Achievement: Multilevel Modelling with Student and Staff/Teacher Responses

Sophie Maxwell^1^, Katherine J Reynolds^2^, Eunro Lee^3*^, Emina Subasic^4^, & Dave Bromhead^5^

^1^School of Education, RMIT University, Australia

^2^Research School of Psychology, Australian National University, Australia

^3^School of Psychological and Clinical Sciences, Charles Darwin University, Australia

^4^ School of Psychology, University of Newcastle, Australia

^5^ Education and Training Directorate, Australian Capital Territory, Australia.

* Correspondence: Eunro Lee, ^3^School of Psychological and Clinical Sciences, Charles Darwin University, Australia , Darwin, NT 0909, Australia Email: eunro.lee@cdu.edu.au

# Supplementary Data:

**Construct Validation of the School Climate and School Identification Measure-Staff (SCASIM-Sf)**

Measuring school climate has been a significant challenge to the research effort, as addressed in the main thesis. The literature has also been criticized for measuring school climate inconsistently and unreliably (Thapa et al., 2013). Various measurement instruments have been used to measure student and staff perceptions. Some of the most widely used instruments are the Organizational Climate Descriptive Questionnaire (Halpin & Croft, 1963), the School Climate Survey (Haynes et al., 1997), Organizational Climate Index (Hoy, Smith, & Sweetland, 2002), and the Organizational Health Inventory (OHI) (Hoy, Tarter & Kottkamp, 1991).

Nevertheless, there have been calls for more psychometrically sound scales which target staff perceptions (Liu, Ding, Berkowitz, & Bier, 2014; Bear et al., 2014), and parallel scales that measure both staff perceptions and student perceptions (Brand, 2008). There has also been interest in measuring school identification (Bizumic et al., 2009) as a psychological mechanism that explains the processes of school climate impact. It is expected that the SCASIM-Sf will meet some of these research requests. This CFA also aims to provide the main study with a reliable and valid measure of staff school climate perception.

The data used for the CFA is staff surveys responses, as part of the ‘school climate’ project described previously. The hypothesized factor structure for the SCASIM-Sf is derived from the SCASIM-St’s factor structure because the staff measure was theoretically developed as parallel to the student version. This structure, briefly described in main thesis, is expected to be verified by using CFA methods, which are the appropriate methods to use when there is a hypothesized factor structure (Thompson, 2004). The respective items of each factor are also examined whether or not they are valid indicators of the respective latent construct.

**Method**

**Procedures and Participants**

A larger sample of the staff survey responses was used for the CFA. See the main study for the procedure for collecting the staff data. 1769 members of staff from 72 public schools in the ACT participated in the survey. 13.3% were administrative staff and 86.7% were teaching staff. 77.7% of the sample was female and 11% did not report their gender. The mean response rate was 55.43% (*SD*= 18.749). Their average teaching experience was 11.95 years (*SD* = 7.99). The average age was 41.08 years old (*SD* = 11.26), but 14.3% of the sample did not report their age.

**Measures**

**Staff perceptions of school climate and school identification.** 36 items are proposed to form four subscales for school climate: shared values and approach (e.g. “students and staff are working towards the same goals”), staff-student relations (e.g. “staff care about students”), staff-staff relations (e.g. “staff value and respect each other”) and academic emphasis (e.g. “teachers set high standards for learning in their classes”). The measure also contains another proposed scale for school identification, in order to test a theoretical model in the main study, which hypothesises that school identification interacts with how school climate comes to affect student outcomes. Staff school identification is measured by six items that have been adapted from the social identification literature to the school climate context (e.g. Bizumic et al., 2009; Haslam, 2004; Turner et al., 2014). These include items such as “being a part of this school is important to me” and “I identify with this school”.

**Analytical Strategy**

After screening and cleaning the data for missing values and outliers, five different CFA models were competitively tested in order to examine the underlying factor structures for the measure. Structural equation modelling (SEM) methods (refer to Arbuckle, 1997 and Bollen, 1989) were employed with MPlus version 7.31 (Muthén & Muthén, 2015), in order to incorporate the measurement models and examine the indicator items and sub-factors in the first and second orders. A proper estimator was chosen based on the normality distribution test of the items.

The structure of the data (staff nested within schools) implied that the assumption of independent observations could be violated (staff responses within each school are expected to be more similar than staff responses between schools). The intra-class correlations (ICCs) and design effects for the 36 items were computed to examine the extent to which the variability in staff responses was accounted for by this nested structure. If ICCs are above zero and the design effects are above two, it suggests that responses within schools are not independent (Hox, 2010) and statistical solutions should be sought with multilevel modelling. Empirically derived modification indices were used and applied to all models to improve model fit based on MPlus results.

Plausible models were tested in order to identify factor structures underlying and causing the 36 item responses. The most parsimonious model was selected out of the five suggested models, based on conventional model fit information and theoretical considerations. Model fit was evaluated with the *χ^2^* statistic, of which significance implies the model does not fit the data.  However, it is sensitive to sample sizes and violations of normality, and will “almost always be significant” (Iacobucci, 2010, p. 91). Therefore alternative model fit indices were used. Model fit was deemed acceptable if the Comparative Fit Index (CFI) and the Tucker-Lewis Index (TLI) were close to or exceeded 0.95, the standardised root mean squared residual (SRMR) was close to or less than 0.08, and the root mean squared error of approximation (RMSEA) was close to or less than 0.06 (Byrne, 1994; MacCallum, Browne, & Sugawara, 1996). Good fit was further implied if the upper limit of the confidence interval of the RMSEA did not exceed 0.08 (Hu & Bentler, 1998).

**Results**

Before conducting the CFA, the data was screened and cleaned using SPSS Version 22.0 to check that the assumptions of multivariate normality and linearity were satisfied. Staff cases with fewer than 10 responses were deleted as tried but incomplete responses and all the rest cases were included without hypothesising any distinct populations from outliers. Descriptive statistics (skewness and kurtosis scores) showed that all items were significantly and negatively skewed, thereby violating the assumption of normality. This abnormal distribution was confirmed by the significant result on the Shapiro-Wilk test (*p* = .000). Subsequently, the MLR estimator was chosen to accommodate non-normal data for the CFAs, accounting for these violations of the assumption of normality, potential heteroscedasticity and missing data.

The results demonstrated the nested dependency in the staff data, where ICC results ranged from 0.04 to 0.24 and design effects ranged from 2.00 to 6.99 (all except one item had design effects over 2). Therefore, a Mplus ‘Type = Complex’ option of analysis was used with the cluster variable of school in order to control the intercorrelations of the staff data in CFAs (Brown, 2014). To estimate the model parameters, maximum likelihood estimation with robust scaling (MLR) was employed.

**Factor structure of the SCASIM-Sf: CFA Model Selection.** Table 1 shows the goodness of fit for the five models tested in the CFAs. They are from the final models after modification indices were applied for each. The first model to be tested was a one-factor model (Model A), with all 36 items loading onto the latent school climate factor. As anticipated, this model was a poor fit. It was also theoretically weak, since it assumed that school climate is a unitary construct, allowing no conceptual distinction between the five hypothesized aspects of school climate.

Model fit improved slightly with the two-factor model (school climate and school identification; Model B). This two-factor model was also more theoretically informed, since school identification and school climate have been confirmed as distinct constructs (Bizumic et al., 2009; Reynolds, Lee, Turner, Bromhead, & Subasic, 2014), wherein school identification is a *process* and school climate is a property of the school (school climate is the *object* of the social identification). However, these first two models did not allow the proposed sub-factors (SfStR, SfsfR, AE and SVA) to correlate with each other and hence, were theoretically inferior to the following three models tested.

Model E was a 5-factor model, with the component items for each proposed subscale loading onto their respective factors and all the five factors are intercorrelated. This showed good model fit. However, it was statistically and theoretically inferior to Model C because it did not conceptualise a general higher order latent ‘school climate’ factor that would accommodate and cause the four highly correlated aspects of school climate.

Model C modelled a second-order factor, school climate, consisting of the four theorised sub-factors of school climate (SSf, SSfR, AE and SVA) (see Figure X). The theoretical distinction between school identification and school climate was also reflected in Model C, with school climate being correlated (but not predicted) by the first order school identification factor. This was the mirrored factor structure to the student scale. As predicted, Model C showed good model fit. In contrast, Model D modelled school identification was a component sub-factor of school climate rather than a correlated factor (less theoretically informed). Model fit indices were, of course, identical for Model C and Model D. The only difference was theoretical, since Model D captured school identification as a mere sub-factor of school climate, while Model C modelled school identification as a correlated factor (suggesting it could be a psychological mechanism or something else). Subsequently, Model C was selected over the other models because it was more theoretically credible and also the most parsimonious.

**Factor loadings of indicator items by measure.** Table 1 and Figure 1 present the factor loadings of the measure items for each sub-factor of the final SCASIM-Sf. Item loadings were all accounted for by their latent sub-factors, and showed valid indicator psychometrics ranging from 0.633 to 0.967 (see Figure 1). The latent school climate construct also accounted for all sub-factors, where the four sub-factors showed excellent second-order loadings ranged from .65 (academic emphasis) to .98 (shared values and approach). The school identification also showed high correlation with the general second-order school climate construct (0.79).

Correlation analysis was conducted to examine the extent to which the subscales were related to one another (Table 3). Results revealed relatively medium to high intercorrelations among latent variables (*r* ranged from .49 to .81, *p* < 0.01; Cohen, 1988, 1992). Finally, the sub-scales were highly internally consistent (see Table 4), as was the entire scale (36 items, α = .98).

**Discussion**

Results from this CFA demonstrate the stable factor structure of the 36-item SCASIM-Sf and its good psychometric properties. The factor structure hypothesized from the EFA and CFA on the student version of the scale (the SCASIM-St) was supported; whereby a second-order, general concept of ‘school climate’, accounted for the correlations between the four theoretically and empirically distinct first-order factors and correlated with a fifth first-order factor, ‘school identification’. The present CFA on the SCASIM-Sf also confirmed that items were adequately loaded to their sub-factors, and were internally consistent.

However, some limitations should be noted. The sample was quite homogenous, with staff members belonging to 72 similar schools in a fairly uniform region of one Australian territory. Future studies could verify the factor structure with a more diverse sample in different educational settings. Future studies could also obtain information about the scale’s convergent, discriminant, concurrent and construct validity by correlating the SCASIM-Sf with existing staff school climate scales and major outcome measures for staff.

The scale fits an influential school climate theory, with subscales mirroring Moos and Moos’ (1978) conceptualisation of three overarching components in school climate; a) relationships, b) personal growth and goal orientation, c) system maintenance and change dimensions (order, values and norms).

This study has been useful by determining the factor structure of the SCASIM-Sf to justify using it as a valid measure in the main study in the thesis. More broadly, the SCASIM-Sf is a useful research tool to measure staff perceptions of school climate and levels of school identification and their stability over time, for identifying issues and challenges in schools and districts and for testing the effectiveness of interventions in a more systematic manner. It would be especially useful when used with the mirroring student SCASIM-St, and these paired scales answer the call for measuring diverse perceptions to get a more complete picture of the health of school climates (Brand et al., 2008; Liu et al., 2014). The paired scales enable comparisons between student and staff perceptions, as largely similar items were found to form largely the same sub-factors. Hence, this CFA has contributed to research efforts by validating a parsimonious and psychometrically sound school climate scale, moving the school climate construct further towards a place of conceptual clarity and practical utility.

# Supplementary Figures and Tables

Table 1.  *Goodness of fit statistics for the alternative factor models the SCASIM-Sf*

*Note:* *χ^2^* = chi square statistic; *df* = degrees of freedom; *N* (para) = number of parameters; CFI = Comparative Fit Index; TLI = Tucker Lewis Index; SRMR = Standardised Square Root Mean Residual; RMSEA = Root Mean Square Error of Approximation; RMSEA C.I. = 90% Confidence interval around RMSEA.

*Indicates *χ^2^* are statistically significant at *p* < .001.

Table 2. *Item loadings for the final SCASIM-Sf*

| Subscale Name | Items | Item loading |
| --- | --- | --- |
| Shared Values and Approach  (SVA) | 1. Students and staff are working towards the same goals | 0.81 |
|  | 1. There is a sense that we are all on the same team | 0.85 |
|  | 1. There is school spirit and pride | 0.86 |
|  | 1. The school’s values and goals are well understood | 0.84 |
|  | 1. New students and staff are made to feel welcome as part of the group | 0.80 |
|  | 1. Student and staff who uphold the values of the school are recognised and celebrated | 0.77 |
|  | 1. The expectations and rules are clear | 0.86 |
|  | 1. The rules related to discipline are clear and well-understood by staff and students | 0.82 |
| Staff to Student Relations  (StSfRel) | 1. Staff care about students | 0.83 |
|  | 1. Staff are friendly to students | 0.88 |
|  | 1. Staff go out of their way to help students | 0.87 |
|  | 1. Staff treat students with respect | 0.90 |
|  | 1. Staff listen to what students have to say most of the time | 0.89 |
|  | 1. Staff involve students in decisions and planning | 0.63 |
|  | 1. Staff are fair in their dealing with students | 0.81 |
|  | 1. Staff show understanding to students | 0.88 |
|  | 1. Staff take students’ concerns seriously | 0.83 |
| Staff to Staff Relations  (SfSfRel) | 1. Staff value and respect each other | 0.72 |
|  | 1. Staff have a consensual approach to managing issues within the school | 0.80 |
|  | 1. The way decisions are made in the school is appropriate | 0.97 |
|  | 1. The decisions made by the school leadership are fair | 0.95 |
|  | 1. The school leadership deserved its position of authority | 0.89 |
| Academic Emphasis  (AE) | 1. Teachers encourage students to try out new ideas (think independently) | 0.77 |
|  | 1. Teachers challenge students to do better | 0.86 |
|  | 1. Teachers are willing to give students extra help on school work if needed | 0.81 |
|  | 1. Teachers set high standards for learning in their classes | 0.87 |
|  | 1. Teachers expect everyone to work hard | 0.85 |
|  | 1. Teachers want every students to do their best | 0.85 |
|  | 1. Teachers believe that every student can be a success | 0.82 |
|  | 8. Teachers give useful feedback | 0.84 |
| School Identification  (SchId) | 1. Being a part of this school is important to me | 0.80 |
|  | 1. I am happy to be a part of this school | 0.87 |
|  | 1. I feel a strong connection with this school | 0.93 |
|  | 1. I identify with this school | 0.93 |
|  | 1. I feel I belong at this school | 0.91 |
|  | 1. I care about this school | 0.80 |

Table 3.

| Subscale | 1 | 2 | 3 | 4 | 5 |
| --- | --- | --- | --- | --- | --- |
| 1. SchId | — |  |  |  |  |
| 1. SVA | 0.71** | — |  |  |  |
| 1. SfSdRel | 0.54** | 0.65** | — |  |  |
| 1. AcaEmp | 0.49** | 0.62** | 0.79** | — |  |
| 1. SfRel | 0.68** | 0.81** | 0.59** | 0.55** | — |

*Correlations between the SCASIM-Sf factors*

*Note. N* = 2280. SchId: School Identification; SVA = Shared values and approach; SfSdRel = Staff-Student Relations; AcaEmp = Academic Emphasis; SfRel = Staff-Staff Relations.

** Correlation is significant at the *p* < 0.01 level (2-tailed).

Table 4.

| Sub-factor/Scale | *Number of items* | $\alpha$ |
| --- | --- | --- |
| School identification | 6 | .95 |
| Shared values and approach | 8 | .95 |
| Staff-student relations | 9 | .95 |
| Staff-staff relations | 5 | .94 |
| Academic emphasis | 6 | .95 |

*Internal Consistency of the Staff Scales*

*Note.* $\alpha$ = Cronbach’s alpha.

## Supplementary Figures

**
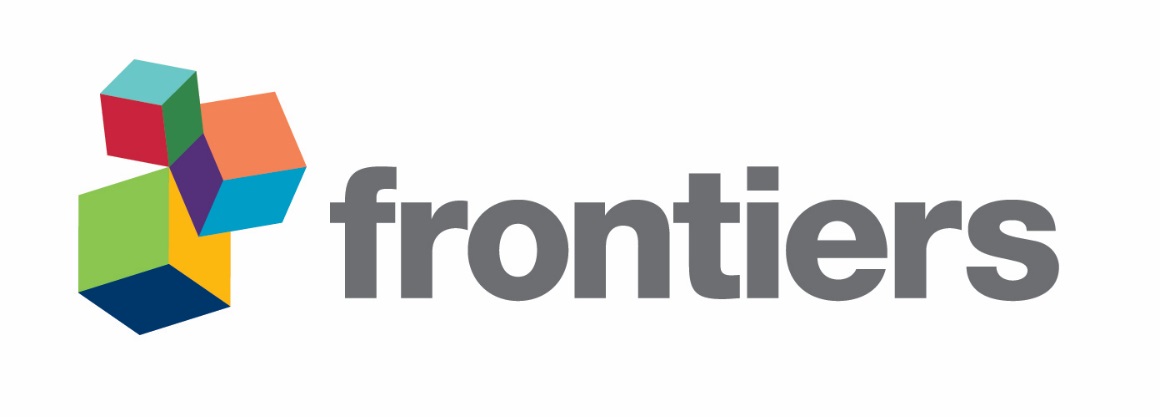
**

**Supplementary Figure 1.** A diagram of the final factor structure of the SCASIM-Sf, with item loadings. *Note.* Error terms and error correlations are omitted for simplicity. SEM visual notation was used, where rectangles are items and ellipses are latent constructs.
